# Loss of Nrf2 abrogates the protective effect of Keap1 downregulation in a preclinical model of cutaneous squamous cell carcinoma

**DOI:** 10.1038/srep25804

**Published:** 2016-05-24

**Authors:** Elena V. Knatko, Maureen Higgins, Jed W. Fahey, Albena T. Dinkova-Kostova

**Affiliations:** 1 Jacqui Wood Cancer Centre, Division of Cancer Research, School of Medicine, University of Dundee, Dundee, DD1 9SY, Scotland, UK; 2 Lewis B. and Dorothy Cullman Chemoprotection Center, Department of Pharmacology and Molecular Sciences, Johns Hopkins University School of Medicine, Baltimore, MD 21205, USA; 3 Center for Human Nutrition, Department of International Health, Johns Hopkins University Bloomberg School of Public Health, Baltimore, MD 21205, USA; 4 Division of Clinical Pharmacology, Department of Medicine, Johns Hopkins University School of Medicine, Baltimore, MD 21205, USA

## Abstract

Cutaneous squamous cell carcinomas (cSCC) are the most common and highly mutated human malignancies, challenging identification of driver mutations and targeted therapies. Transcription factor NF-E2 p45-related factor 2 (Nrf2) orchestrates a cytoprotective inducible program, which counteracts the damaging effects of solar UV radiation, the main etiological factor in cSCC development. Downregulation of Kelch-like ECH-associated protein 1 (Keap1), a Cullin-3/Rbx1 ubiquitin ligase substrate adaptor protein, which mediates the ubiquitination and proteasomal degradation of Nrf2, has a strong protective effect in a preclinical model of cSCC. However, in addition to Nrf2, Keap1 affects ubiquitination of other proteins in the carcinogenesis process, including proteins involved in inflammation and DNA damage repair. Here, we generated Keap1^*flox/flox*^ SKH-1 hairless mice in which Nrf2 is disrupted (Keap1^*flox/flox*^/Nrf2^−/−^) and subjected them chronically to solar-simulated UV radiation. We found that the incidence, multiplicity and burden of cSCC that form in Keap1^*flox/flox*^/Nrf2^−/−^ mice are much greater than in their Keap1^*flox/flox*^/Nrf2^+/+^ counterparts, establishing Nrf2 activation as the protection mediator. Our findings further imply that inhibition of Nrf2 globally, a strategy proposed for cancer treatment, is unlikely to be beneficial.

Cutaneous squamous cell carcinomas (cSCC) are among the most frequent and highly mutated human cancers, carrying one mutation per ~30,000 bp of coding sequence^1^. Such astonishingly high burden of genetic alterations presents a major challenge in identifying driver mutations and developing targeted therapies, highlighting the importance of cancer prevention strategies. Solar ultraviolet (UV) radiation, the most abundant carcinogen in our environment, is the main factor in the etiology of human cSCC^2^. Public awareness campaigns regarding general sun avoidance have not shown significant benefits – indeed, the incidence of cSCC is steadily rising, and the development of new strategies for prevention against know risk factors are urgently needed.

In sun-exposed skin, UV radiation causes DNA damage, inflammation, and immunosuppression, which collectively contribute to the development of cSCC^2^. Many of the damaging processes caused by solar UV radiation are counteracted by transcription factor NF-E2 p45-related factor 2 (Nrf2, gene name *NFE2L2*), which orchestrates a broadly cytoprotective transcriptional program allowing adaptation and survival under conditions of electrophilic, oxidative, and inflammatory stress^3^,^4^,^5^. Under basal conditions, Nrf2 undergoes continuous ubiquitination and proteasomal degradation, which is primarily mediated by Kelch-like ECH-associated protein 1 (Keap1)^6^, a Cullin-3/Rbx1 ubiquitin ligase substrate adaptor protein^7^,^8^,^9^. Specific reactive cysteine residues within Keap1 serve as sensors for a variety of endogenous and exogenous sulfhydryl-reactive small molecules (termed inducers)^10^,^11^, which inhibit the substrate adaptor function of Keap1, leading to Nrf2 activation, and have shown protective effects in numerous animal models of disease, including cSCC^12^,^13^.

We have recently developed a mouse model of solar-simulated UV (SSUV) radiation-induced cSCC, in which the murine tumors closely resemble the histopathological spectrum of human cSCC^13^. In this model, the incidence, multiplicity and burden of cSCCs that form in SKH-1 hairless mice, in which Nrf2 is genetically constitutively activated by downregulation of Keap1 (Keap1^*flox/flox*^), are lower than those that arise in their wild-type counterparts^13^. Keap1^*flox/flox*^ mice have two floxed alleles of the *keap1* gene, which reduces its expression and consequently increases the levels of Nrf2 and its downstream target genes^13^,^14^, suggesting that the protective effect of Keap1 downregulation against SSUV skin damage and photocarcinogenesis are due to altered expression of Nrf2 target genes. However, alternative possibilities should be also considered. Thus, in addition to Nrf2, Keap1 binds and promotes the ubiquitination and/or degradation of other proteins, such as IκB kinase β (IKKβ), a protein kinase implicated in tumor development through activation of the nuclear factor (NF)-κB pathway^15^. Moreover, Keap1 binds and mediates the ubiquitination of PALB2^16^,^17^, leading to suppression of the assembly of the BRCA1-PALB2-BRCA2 protein complex, which plays a key role in DNA double strand break repair by homologous recombination^17^. Thus, it was important to ask whether or not Nrf2 is the mediator of the protective effect of the Keap1 downregulation that was observed in the SSUV-induced skin carcinogenesis model. To answer this question, we generated Keap1^*flox/flox*^ SKH-1 hairless mice in which Nrf2 was disrupted (Keap1^*flox/flox*^/Nrf2^−/−^ SKH-1 hairless mice). We found that loss of Nrf2 completely abrogates the protective effect of Keap1 downregulation, thus establishing Nrf2 activation as the mediator of protection in this preclinical model. Taken together with previous reports on the chemopreventive effects of pharmacological activators of Nrf2 against skin photodamage and photocarcinogenesis, our findings imply that mild Nrf2 activation is an effective strategy for protection against SSUV radiation-induced cSCC.

## Materials and Methods

### Animals

The animal experiments were performed according to the regulations described in the UK Animals (Scientific Procedures) Act 1986 and in strict compliance with institutional guidelines. The animal study plan was developed after ethical approval (Project Licence 60/5986), and was further approved by the Named Veterinary Surgeon and the Director of Biological Services of the University of Dundee. Keap1^*flox/flox*^ SKH-1 hairless mice, in which Nrf2 is disrupted (i.e., Keap1^*flox/flox*^/Nrf2^−/−^), were generated by crossing Keap1^*flox/flox*^/Nrf2^+/+^ and Keap1^+/+^/Nrf2^−/−^ mice, both on the SKH-1 hairless genetic background^13^. The mice were bred and maintained in our facility with free access to water and food (pelleted RM1 diet from SDS Ltd., Witham, Essex, UK), on a 12-h light/ 12-h dark cycle, 35% humidity. All experimental animals were age-matched and female. For biochemical analysis, the mice were euthanized, their dorsal skin was harvested, and immediately frozen in liquid N_2_. Frozen skin was pulverized into powder under liquid N_2_, and stored at −80 °C until analysis.

### Immunoblotting

Pulverized skin powder (~30 mg) was resuspended and homogenized in ice-cold radioimmunoprecipitation assay buffer (50 mM Tris-HCl, pH 7.5; 150 mM NaCl; 1% NP-40; 0.5% sodium deoxycholate; 0.1% sodium dodecyl sulfate; 1 mM ethylenediaminetetraacetic acid [EDTA]), containing protease inhibitors (Roche). The samples were mixed with LDS loading buffer (Invitrogen), loaded (35 μg of protein/lane) on gradient (4–12%) Bis-Tris NuPAGE gel, and run under reducing condition using MOPS running buffer, with SeeBlue pre-stained molecular weight markers (Invitrogen) for size reference. The separated proteins were electrophoretically transferred to a nitrocellulose membrane (Amersham Protran 0.45 NC). The membrane was then cut into three parts: the top part was probed for Nrf2, the middle part, for Keap1, and the bottom part, for β-actin. Parallel samples were processed in an identical way for detection of PALB2 levels, but the membrane was cut into two parts: the top part was probed for PALB2, and the bottom part, for β-actin. After blocking with 5% non-fat milk at 4 °C for 2 h, immunoblotting was performed using the following antibodies: anti-Nrf2 (rabbit monoclonal, 1:1000 dilution, Cell Signaling), anti-Keap1 (rabbit polyclonal, 1:2000 dilution, a kind gift from John D. Hayes, University of Dundee^18^), or anti-PALB2 (rabbit polyclonal, 1:1000 dilution, Sigma-Aldrich Co.). The immunoblot for β-actin (Sigma–Aldrich Co., 1:10000 dilution) served as a loading control.

### Enzyme activity assays

Pulverized skin powder (~30 mg) was resuspended in ice-cold phosphate buffer (100 mM potassium phosphate, pH 7.4; 100 mM KCl; 0.1 mM EDTA), and homogenized in an ice bath. The insoluble material was removed by centrifugation at 4 °C at 15,000 × *g* for 10 min. The supernatant fractions were used to determine the enzyme activities of NAD(P)H:quinone oxidoreductase 1 (NQO1) using menadione as a substrate^19^ and of glutathione *S*-transferase (GST) using 1-chloro-2,4-dinitrobenzene (CDNB) as a substrate^20^. The bicinchoninic acid (BCA) assay (Thermo Scientific) was employed to measure the protein concentration in each supernatant fraction, which was used to calculate the specific enzyme activity.

### Quantitative real-time PCR

RNA was extracted from pulverized skin powder using RNeasy Fibrous Tissue Kit (Qiagen Ltd.). RNA (500 ng) was reverse transcribed into cDNA using Omniscript RT Kit (Qiagen Ltd.). The mRNA levels for Keap1 were determined by quantitative real-time PCR on a Perkin Elmer/Applied Biosystems Prism Model 7700 Sequence Detector instrument using primers and probe (TaqMan^®^ Gene Expression Assays) purchased from Life Technologies. The TaqMan data for the mRNA species were normalized using β-actin (mouse ACTB, 4352933E) as an internal control.

### Cutaneous carcinogenesis

The animals were individually marked and housed in such a way that every cage contained representatives of each genotype. Cutaneous carcinogenesis was initiated when the mice were 8-week old by subjecting the animals chronically twice a week (on Tuesdays and Fridays) for 15 weeks to SSUV radiation (comprised of 2 J/cm^2^ UVA and 90 mJ/cm^2^ UVB). The irradiation unit (Daavlin, Bryan, OH) was equipped with an electrical fan to prevent excessive heating. For exposure to UV radiation, the animals were placed in clear, bedding-free cages. SSUV radiation was provided by UVA340 lamps (Q-Lab, Germany), which simulate the solar UV radiation in the critical short wavelength region, from 365 nm to the solar cutoff of 295 nm, and have a peak emission at 340 nm. A UVB Daavlin Flex Control Integrating Dosimeter was used to quantify the radiant dose. The dose was confirmed by use of an external radiometer (X-96 Irradiance Meter; Daavlin, Bryan, OH) before and after each irradiation session. Tumors (defined as lesions ≥1 mm in diameter) were recorded once a week. To calculate the tumor volumes (v = 4πr^3^/3), the height, length, and width of each tumor were measured, and the average of the three measurements was used as the diameter.

### Statistical analysis

Statistical analyses were performed using either Excel (Microsoft Corp.) or Stata 11.2 (Statacorp, College Station, TX, USA). Values are means ± 1 S.D. or 1 S.E.M., as indicated in the figure legends.

## Results and Discussion

**Figure 1:**
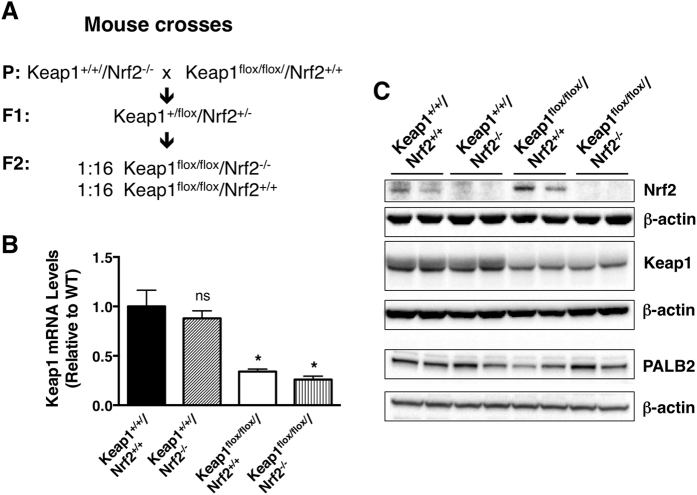
Levels of Nrf2 and Keap1 in the skin of female Keap1^*flox/flox*^/Nrf2^+/+^ and Keap1^*flox/flox*^/Nrf2^−/−^ SKH-1 hairless mice. (**A**) Breeding scheme used to generate Keap1^*flox/flox*^/Nrf2^+/+^ and Keap1^*flox/flox*^/Nrf2^−/−^ SKH-1 hairless mice. (**B**) RNA was extracted from pulverized powder obtained from skin samples isolated from mice (female, 12–15-week-old, n = 3) of each of the four genotypes. After reverse transcription into cDNA, the mRNA levels for Keap1 were determined by quantitative real-time PCR using β-actin as an internal control. Statistical analysis was performed using Excel (Microsoft). The differences between groups were determined by Student’s t test. Values are means ± 1 S.D. Each mutant genotype was compared to the wild-type (Keap1^+/+^/Nrf2^+/+^) genotype. ns, non-significant; *p < 0.01. (**C**) Pulverized murine skin powder (~30 mg) was homogenized, and proteins (35 μg) were separated by electrophoresis, transferred to nitrocellulose membrane, and probed for Nrf2, Keap1, PALB2, and β-actin. Each lane represents a sample from an individual 12-week-old female mouse of the indicated genotype. The full-size blots are shown in Supplementary Fig. 1.

We generated Keap1^*flox/flox*^ SKH-1 hairless mice in which Nrf2 was disrupted (i.e., Keap1^*flox/flox*^/Nrf2^−/−^) by crossing Keap1^*flox/flox*^/Nrf2^+/+^ and Keap1^+/+^/Nrf2^−/−^ mice, both on the SKH-1 hairless genetic background (Fig. 1A). The Keap1^*flox/flox*^ mice were originally developed to allow for generating tissue-specific Keap1^−/−^ mice, but were found to have lower expression of Keap1 (i.e., hypomorphic *keap1* alleles) and consequently, increased levels of Nrf2 and expression of its target genes in all tissues^13^,^14^. Thus, the Keap1^*flox/flox*^ mice represent a genetic animal model for global constitutive Nrf2 activation to levels which are comparable to the levels that can be achieved by administration of small-molecule pharmacological Nrf2 activators.

**Figure 2:**
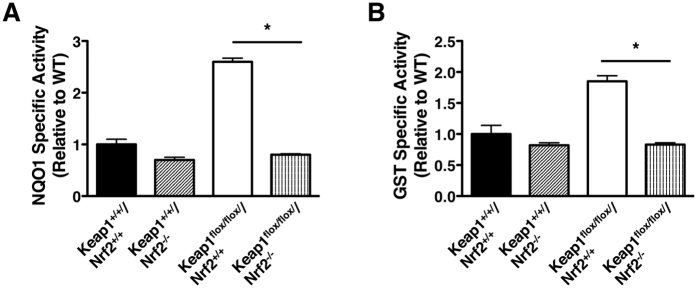
Cutaneous enzyme activity of the Nrf2 transcriptional targets NQO1 and GST. Pulverized murine skin powder (~30 mg) obtained from skin samples isolated from animals (female, 12–15-week-old, n = 3) of each of the four genotypes was resuspended in ice-cold potassium phosphate buffer, homogenized in an ice bath, and subjected to centrifugation at 4 °C (15,000 x g for 10 min). The specific enzyme activities of NQO1 (with menadione as a substrate) (**A**) and GST (CDNB as a substrate) (**B**) were determined in supernatant fractions. Statistical analysis was performed using Excel (Microsoft). The differences between groups were determined by Student’s *t* test. Values are means ± 1 S.D. *p < 0.001.

Consistent with previous observations, the cutaneous mRNA levels for Keap1 are comparable in wild-type (Keap1^+/+^/Nrf2^+/+^) and Nrf2-knockout (Keap1^+/+^/Nrf2^−/−^) mice, but these levels are reduced by 70% in Keap1^*flox/flox*^/Nrf2^+/+^ (p < 0.01) and Keap1^*flox/flox*^ /Nrf2^−/−^ (p < 0.01) mice (Fig. 1B). The differences in the Keap1 mRNA levels among the four genotypes are in agreement with the differences in the corresponding protein levels, which are ~60% lower in Keap1^*flox/flox*^/Nrf2^+/+^ and Keap1^*flox/flox*^ /Nrf2^−/−^ mice in comparison with Keap1^+/+^/Nrf2^+/+^ and Keap1^*+/+*^/Nrf2^−/−^ animals (Fig. 1C). As expected, Keap1^*flox/flox*^/Nrf2^−/−^ mice have no detectable Nrf2 protein levels in their skin, whereas the presence of the transcription factor is readily detectable in skin isolated from Keap1^*flox/flox*^/Nrf2^+/+^ animals (Fig. 1C). In contrast to the increased protein levels of Nrf2 in Keap1^*flox/flox*^/Nrf2^+/+^ mice in comparison with Keap1^+/+^/Nrf2^+/+^ animals, there were no consistent differences among the genotypes in the levels of PALB2 (Fig. 1C), a protein which functions in homologous recombination. This is in agreement with the reported role of Keap1 in mediating the ubiquitination, but not the degradation of PALB2^16^,^17^. In concordance with the differences in the Nrf2 levels between the Keap1^*flox/flox*^/Nrf2^+/+^ and Keap1^*flox/flox*^/Nrf2^−/−^ genotypes, the specific activity of the Nrf2-dependent enzymes NAD(P)H:quinone oxidoreductase 1 (NQO1) (Fig. 2A) and glutathione *S*-transferase (GST) (Fig. 2B) are lower in Keap1^*flox/flox*^/Nrf2^−/−^ compared to Keap1^*flox/flox*^/Nrf2^+/+^ skin by 70% (p < 0.001) and 55% (p < 0.001), respectively.

**Figure 3:**
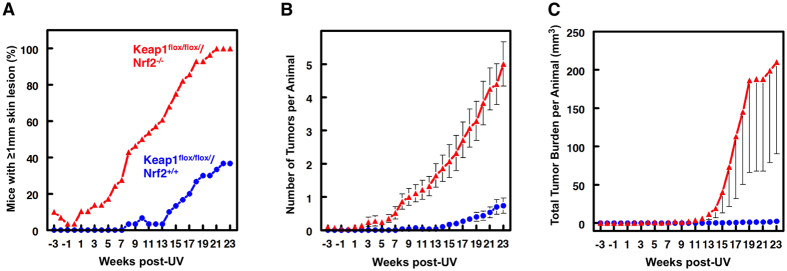
Loss of Nrf2 abrogates the protective effect of Keap1 downregulation against cutaneous carcinogenesis mediated by SSUV radiation. Female Keap1^*flox/flox*^/Nrf2^+/+^ and Keap1^*flox/flox*^/Nrf2^−/−^ SKH-1 hairless mice (n = 30, with every cage housing representatives of each genotype) were exposed chronically to SSUV radiation (comprised of 2 J/cm^2^ UVA and 90 mJ/cm^2^ UVB) beginning at 8 weeks of age, twice a week for 15 weeks. During the subsequent 23 weeks after the end of the irradiation schedule, the appearance of tumors was monitored weekly, and lesions ≥1 mm in diameter were mapped, counted, and their volumes determined. The graphs show: tumor incidence (the number of mice with tumors) (**A**), multiplicity (the number of tumors per mouse) (**B**), and the total tumor burden (the sum of the volumes of all tumors per mouse) (**C**), all expressed as average values ± S.E.M. based on the total number of animals at risk. Statistical analysis was performed using Stata 11.2 (Statacorp, College Station, TX, USA).

Having established the differences in Nrf2 and the expression of its downstream target genes between the two mouse lines, we subjected groups of 30 mice of each genotype to chronic sub-erythemal doses of SSUV radiation, twice a week for 15 weeks. The development of cSCC was monitored during the subsequent 23 weeks. Consistent with our previous report^13^, the Keap1^*flox/flox*^/Nrf2^+/+^ mice were remarkably protected against the carcinogenic effects of SSUV radiation, and only 40% of the animals had skin lesions ≥1 mm in diameter at termination of the experiment (23 weeks after the SSUV radiation schedule was discontinued) (Fig. 3A). The protective effect of Keap1 downregulation was completely lost in the absence of Nrf2, and 100% of the Keap1^*flox/flox*^/Nrf2^−/−^ mice had tumors at that time point, with 50% of them developing their first tumor at week 10 after receiving the last dose of SSUV radiation.

Kaplan–Meier ‘survival analysis’ applied to freedom-from-tumors, followed by a stratified log-rank test for equality of survivor function showed a highly significant difference between the Keap1^*flox/flox*^/Nrf2^+/+^ and Keap1^*flox/flox*^/Nrf2^−/−^ groups (χ^2^ = 14.5; p < 0.0001). The effect of the loss of functional Nrf2 on tumor multiplicity was also evident, and there was a ~5-fold increase, from 0.9 tumors per mouse in the Keap1^*flox/flox*^/Nrf2^+/+^ group to 4.6 tumors per mouse in the Keap1^*flox/flox*^/Nrf2^−/−^ group (Fig. 3B). The difference between the two groups was highly significant by ANOVA followed by Bartlett’s test for equal variances (F 179, p < 0.0001). The Keap1^*flox/flox*^/Nrf2^−/−^ group became significantly different from the Keap1^*flox/flox*^/Nrf2^+/+^ group by week six post-SSUV radiation. In addition to the differences in tumor incidence and multiplicity, the tumor volume (expressed in mm^3^) per mouse was also profoundly affected by the genotype (Fig. 3C), and there was a remarkable ~30–150-fold difference in the total tumor burden between the Keap1^*flox/flox*^/Nrf2^−/−^ and the Keap1^*flox/flox*^/Nrf2^+/+^ groups over the last 9 weeks of the experiment (i.e., weeks 14–23 post-SSUV) (F 21.7, p < 0.0001 by ANOVA with the Scheffe multiple comparison test).

The results from the current study clearly establish that the protective effect of downregulation of Keap1 in the SSUV-mediated cSCC mouse model is due to activation of Nrf2 and encourage the continued development of pharmacologic activators of Nrf2 as chemopreventive agents against photodamage and photocarcinogenesis. Importantly, the magnitude of Nrf2 activation in the Keap1^*flox/flox*^/Nrf2^+/+^ mice is relatively modest (~2-fold) and comparable to the degree of activation of the transcription factor by pharmacological agents in the skin of mice and humans^13^,^21^,^22^, and neither the Keap1^*flox/flox*^/Nrf2^+/+^ nor the Keap1^*flox/flox*^/Nrf2^−/−^ mice had any obvious skin abnormalities. This is in contrast to transgenic mice expressing keratinocyte-specific constitutively active mutant Nrf2 under the control of a β-actin promoter and a CMV enhancer, in which the magnitude of upregulation of Nrf2 target genes is more than 10-fold (and for some genes, even approaching 100-fold), and which display sebaceous gland hypertrophy, hyperkeratosis and cyst formation^23^. The critical importance for cancer prevention by a mild (as opposed to robust) degree of activation of Nrf2 is evidenced by an increasing number of studies from several independent groups of investigators. Thus, it has been shown that keratinocyte-specific robust constitutive activation of Nrf2, which lacks the Keap1-binding domain, promotes HPV8-induced skin papilloma formation in mice^24^, whereas mild pharmacological activation is protective against skin photodamage and photocarcinogenesis^13^,^22^,^25^.

This notion is further supported by the seemingly paradoxical findings that, contrary to its role in cancer prevention, Nrf2 is frequently activated in established human tumors, and contributes to resistance to chemotherapy and radiation therapy. A comprehensive genomic characterization by the Cancer Genome Atlas Research Network has identified mutations in *NRF2, KEAP1*, or *CUL3* in 34% of 178 lung squamous cell carcinomas^26^. Under conditions of oncogenic stress, such as that during sustained activation of KRAS, BRAF, or PI3K-AKT signaling, activation of Nrf2 facilitates cell proliferation^27^,^28^. Notably, in all of these cases the levels of Nrf2 and its classical target genes are constitutively very high, in sharp contrast to the mild activation, which is observed under conditions of Keap1 downregulation or treatment with pharmacological inducers. Taken together, these findings imply that the magnitude and duration of Nrf2-mediated transcriptional responses are critical determinants of the balance between the beneficial and detrimental consequences of Nrf2 activation.

Our results have additional implications for cancer prevention and treatment. Since the discovery of Nrf2, accumulating experimental evidence has clearly demonstrated that: (i) Nrf2-knockout mice are more susceptible than their wild-type counterparts to the toxic and carcinogenic effects of many agents^29^,^30^,^31^, (ii) mild genetic or pharmacological activation of Nrf2 is protective against tumor initiation^13^,^29^, and (iii) Nrf2 deletion increases cancer risk^29^,^31^. Based on these findings, the development of Nrf2 inducers is an attractive strategy for cancer prevention. This is especially important because many Nrf2 inducers, such as isothiocyanates, flavonoids, and carotenoids are phytochemicals present in edible plants and have been consumed for centuries as components of the human diet^32^,^33^,^34^. More recently however, because of the high level of Nrf2 activity in many human tumors, the development of Nrf2 inhibitors has been proposed as a strategy for cancer treatment. Whereas this approach has shown promise initially in animal models as exemplified by the quassinoid phytochemical, brusatol^35^, the interpretation of the inhibitory effects on tumor development in these models is complicated by the fact that, in addition to Nrf2, compounds such as brusatol have the ability to affect fundamental cellular processes, including global protein translation^36^. Several known environmental carcinogens, such as the food contaminant ochratoxin A, have been shown to inhibit Nrf2^37^,^38^,^39^. Moreover, the antioxidants vitamin E and *N*-acetylcysteine, suppress the activity of Nrf2 whilst promoting tumor development and metastasis^40^,^41^. Together with the results from our study, these findings imply that inhibition of Nrf2 globally is unlikely to be beneficial.

## Additional Information

**How to cite this article**: Knatko, E. V. *et al*. Loss of Nrf2 abrogates the protective effect of Keap1 downregulation in a preclinical model of cutaneous squamous cell carcinoma. *Sci. Rep.* **6**, 25804; doi: 10.1038/srep25804 (2016).

## Supplementary Material

Supplementary Information
