# Supplementary material for: Loss of Nrf2 abrogates the protective effect of Keap1 downregulation in a preclinical model of cutaneous squamous cell carcinoma
Source: Sci Rep. 2016 May 24;6:25804. doi: 10.1038/srep25804 (PMC4877584; doi:10.1038/srep25804)
Supplement: Supplementary Information [file srep25804-s1.pdf]

Supplementary Material

Loss of Nrf2 abrogates the protective effect of Keap1 downregulation in a preclinical model of cutaneous squamous cell carcinoma

Elena V. Knatko, Maureen Higgins, Jed W. Fahey and Albena T. Dinkova-Kostova

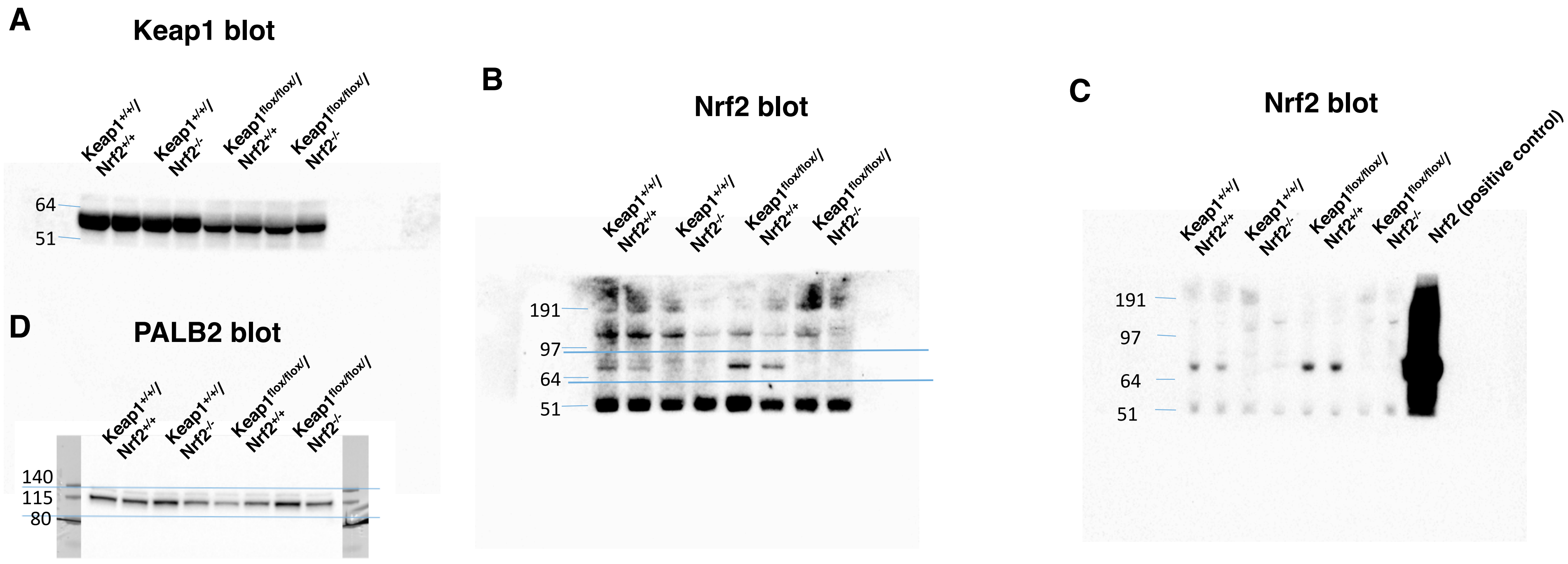

Supplementary Figure 1. Full size blots for the cropped images shown in Figure 1. **(A)** Keap1 blot. **(B)** Nrf2 blot. **(C)** An independent Nrf2 blot which also includes a positive control (lysate of Nrf2-knockout mouse embryonic fibroblasts ectopically expressing Nrf2). **(D)** PALB2 blot.
